# Crocin Exhibits Antitumor Effects on Human Leukemia HL-60 Cells In Vitro and In Vivo

**DOI:** 10.1155/2013/690164

**Published:** 2013-03-19

**Authors:** Yan Sun, Hui-Juan Xu, Yan-Xia Zhao, Ling-Zhen Wang, Li-Rong Sun, Zhi Wang, Xiu-Fang Sun

**Affiliations:** ^1^Department of Pediatric Hematology, The Affiliated Hospital of Medical College, Qingdao University, No. 16 Jiangsu Road, Qingdao 266003, China; ^2^Department of Pharmacy, The Affiliated Hospital of Medical College, Qingdao University, Qingdao 266003, China; ^3^Department of Clinical Laboratory, The Affiliated Hospital of Medical College, Qingdao University, Qingdao, 266003, China

## Abstract

Crocin is a carotenoid of the saffron extract that exhibits antitumor activity against many human tumors. However, the effects of crocin on HL-60 cells in vivo have not been evaluated. This study aimed to examine the effects of crocin on HL-60 cells in vitro and in vivo and investigate the underlying mechanisms. HL-60 cells were treated by crocin, and cell proliferation, apoptosis, and cell cycle profiles were examined by MTT assay, AO/EB staining, and flow cytometry, respectively. Furthermore, HL-60 cells were xenografted into nude mice and treated by crocin, the tumor weight and size were calculated, and the expression of Bcl-2 and Bax in xenografts was detected by immunohistochemical staining. The results showed that crocin (0.625–5 mg/mL) inhibited HL-60 cell proliferation and induced apoptosis and cell cycle arrest at G0/G1 phase, in a concentration and time-dependent manner. In addition, crocin (6.25, 25 mg/kg) inhibited the tumor weight and size of HL-60 xenografts in nude mice, inhibited Bcl-2 expression, and increased Bax expression in xenografts. In summary, crocin inhibits the proliferation and tumorigenicity of HL-60 cells, which may be mediated by the induction of apoptosis and cell cycle arrest and the regulation of Bcl-2 and Bax expression.

## 1. Introduction

Survival rates of children with acute lymphoblastic leukemia (ALL) and acute myeloid leukemia (AML) currently range from 83% to 94% and 60% to 65%, respectively [1]. The survival rates have improved remarkably over the past decades, largely due to conventional chemotherapy. However, the side effects of cytotoxic chemotherapy remain significant. Further improvements in outcomes will depend on anticancer drugs with high efficacy and low toxicity.

*Crocus sativus* L., commonly known as saffron, is a perennial stemless herb of the large Iridaceae family and has been used in cancer therapy [2]. Crocin, a main water-soluble carotenoid of the saffron extract, exhibits anti-tumor activity against many human tumors, such as colorectal, pancreatic, and bladder cancer [3]. Notably, crocin significantly inhibits the growth of cancer cells but has no effects on normal cells [4]. These studies provide strong evidence that crocin has high anti-tumor activity and low cytotoxicity.

It has been reported that carotenoids from saffron were effective in inhibiting the proliferation of HL-60 cells [5]. However, the effects of crocin on HL-60 cells in vivo have not been evaluated, and the mechanism responsible for the antileukemia effects of saffron remains elusive. In the present study, a series of experiments were performed to examine the effects of crocin on HL-60 cells in vitro and in vivo and investigate the underlying mechanisms.

## 2. Materials and Methods

### 2.1. Cell Line and Treatment

Human leukemia HL-60 cells were gifted from the Institute of Hematology and Blood Diseases Hospital, Chinese Academy of Medical Sciences, Tianjin. HL-60 cells were cultured in RPMI-1640 medium (Gibco) supplemented with 10% heat-inactivated fetal bovine serum (FBS) in a humidified incubator of 5% CO2 at 37°C. Crocin was purchased from Sigma (CAS Number 42553-65-1) and diluted in 10 mmol/L phosphate-buffered saline for the appropriate concentration upon used.

### 2.2. Cell Proliferation Assay

Cell proliferation was determined by using MTT assay. Briefly, HL-60 cells were treated with crocin (0.625–10 mg/mL) for 24 h or 48 h. Then the cells were incubated with MTT solution (5 mg/mL in PBS, Sigma) for 4 h and solubilized with DMSO (150 *μ*L). The absorption was measured at 570 nm in an ELISA reader. The following formula was used for the calculation: cell inhibition rate (%) = [1 − (*A* value of the experimental samples/*A* value of the control)] ×  100%.

### 2.3. AO/EB Staining

AO/EB staining of HL-60 cells was performed to detect the apoptotic and necrotic patterns as described previously [6]. Briefly, HL-60 cells (2 × 10^5^ cells/mL) were treated by crocin (0.625, 1.25, 2.5, 5.0, and 10 mg/mL) for 24 h or 48 h and then washed three times with phosphate-buffered saline (PBS). The cells were stained with 100 *μ*g/mL AO/EB for 5 min. At least 200 cells were observed under a fluorescence microscope. The cells were classified as follows: viable, apoptotic, or necrotic. The percentage of apoptotic cell was then calculated by the formula: percentage of apoptotic cell (%) = (amount of apoptotic cell/total cell examined) × 100%.

### 2.4. Cell Cycle Analysis

HL-60 cells were treated with different concentrations of crocin. After 48 h, cells were harvested and fixed in 70% ethanol at 4°C overnight. Fixed cells were stained with 5 *μ*L PI for 20 min on ice in the dark. Finally, the fluorescence emitted by PI-DNA complex was examined at 488 nm. The percentages of cells in various phases of the cell cycle, namely, G0, G1, S, and G2/M, were assessed using a flow cytometry and analyzed by Cell Quest software. 

### 2.5. Animal Xenograft Model

A total of 32 males BALB/c nude mice (3 weeks old) were purchased from Shanghai Laboratory Animal Center, Chinese Academy of Sciences. Animals were maintained under standardized, sterilized conditions (25 ± 2°C, 60–70% relative humidity, 12 hours dark/light cycle) in specific pathogen-free (SPF) laboratory, and were fed a regular nude mice chow. The mice were acclimatized to the housing condition for 1 week. All the experiments were conducted under the guidelines of laboratory animal use and care of the European Community (EEC Directive of 1986; 86/609/EEC).

 Nude mice xenograft models were established by injecting HL-60 cells (1 × 10^7^/0.2 mL) subcutaneously on the back of the right shoulder of each mouse (4 weeks old). Immediately after the injection of HL-60 cells, the nude mice were randomly divided into 4 groups (*n* = 8): control group was treated with 0.2 mL saline/d by daily intraperitoneal injection (i.p. qd); 3 experimental groups were treated with 6.25, 25, and 100 mg/kg crocin (diluted in saline to 0.2 mL, i.p. qd) for 28 days, respectively. Tumor formation time was recorded as the time from injecting HL-60 cells to forming tumor (diameter 5 mm∗5 mm). Tumor formation rate was calculated as the numbers of mice forming tumor/the total numbers of each group × 100%. The tumor volume and body weight were monitored daily throughout the experiments. Tumor volumes were measured by a digital caliper and calculated according to the following formula: tumor volume (mm^3^) = 0.4 × *L* × *W* ^2^; *L* and *W* were the major and minor dimensions of the tumor, respectively [7]. The change ratio of tumor volume was calculated using the formula: (*V*_*n*_ − *V*_0_)/*V*_0_  ×  100%. *V*_*n*_ represented the tumor volume on the *n*th day after injecting HL-60 cells, and *V*_0_ represented the initial tumor volume (diameter 5 mm∗5 mm). The animals were sacrificed at the end of the experiment, and none of them died during the experiments.

### 2.6. Immunohistochemical Analysis

The immunohistochemical staining of Bcl-2 and Bax in the tumor tissue was performed using the streptavidin-biotin-complex peroxidase kit (Boster, Wuhan, China). Finally, the slides were washed, dehydrated, and mounted for microscopic examination and enumeration immunoreactive cells (yellow to brown). Analysis of immunostaining in xenografts was done on a Media Cybernetics Image-Pro Plus analysis system linked to an Olympus microscope. The cells stained positive for Bcl-2 and Bax were quantified by counting the yellow to brown cells and the total number of cells at five randomly selected fields at 400x magnification.

### 2.7. Western Blot Analysis

The tumor tissues were collected and lysed in radioimmunoprecipitation assay (RIPA) buffer supplemented with protease inhibitors. The protein concentrations of the lysate were quantified using the bicinchoninic acid (BCA) protein assay kit (Beyotime Institute of Biotechnology, China). Equal amounts of protein were separated by 10% sodium dodecyl sulfate-polyacrylamide gel electrophoresis (SDS-PAGE) and transferred to polyvinyli-dene fluoride (PVDF) membranes (Bio-Rad, Hercules, CA, USA). Membranes were blocked with PBST (PBS with 0.05% Tween-20) containing 5% nonfat dry milk for 1 h and then incubated at 4°C overnight with Bcl-2, Bax, or *β*-actin antibody (Sigma) in fresh blocking buffer. Membranes were then washed with PBST, incubated with horseradish peroxidase-conjugated secondary antibody (Santa Cruz Biotechnology, Santa Cruz, CA, USA) for 1 h, and developed with the ECL western blotting system. Protein levels were normalized to *β*-actin.

### 2.8. Statistical Analysis

Data were presented as the mean ± standard deviation (SD) and analyzed by one-way analysis of variance (ANOVA) followed by LSD test using the SPSS 17.0 software. Statistical significance of tumor formation rate was assessed with Fisher's exact probability test. Significant differences were defined as *P* < 0.05.

## 3. Results

### 3.1. Crocin Inhibits the Proliferation of HL-60 Cells

**Figure 1:**
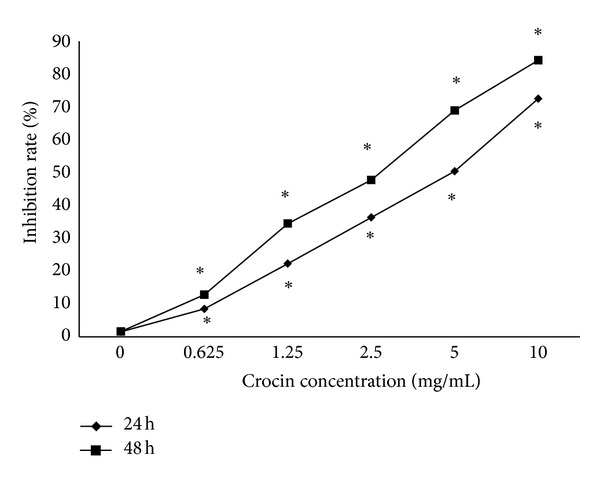
Crocin inhibits the proliferation of HL-60 cells in a dose-and time-dependent manner. HL-60 cells were treated by crocin at the indicated concentration for 24 or 48 h, and the inhibition rate of proliferation was calculated based on MTT assay. *P < 0.05 versus control.

MTT assay showed that compared with the control group, crocin at the various concentrations (0.625–10 mg/mL) significantly inhibited HL-60 cell proliferation, and the inhibitory effect of crocin on HL-60 cell proliferation was dose and time dependent (Figure 1). 

### 3.2. Crocin Induces Apoptosis and Cell Cycle Arrest of HL-60 Cells

**Figure 2:**
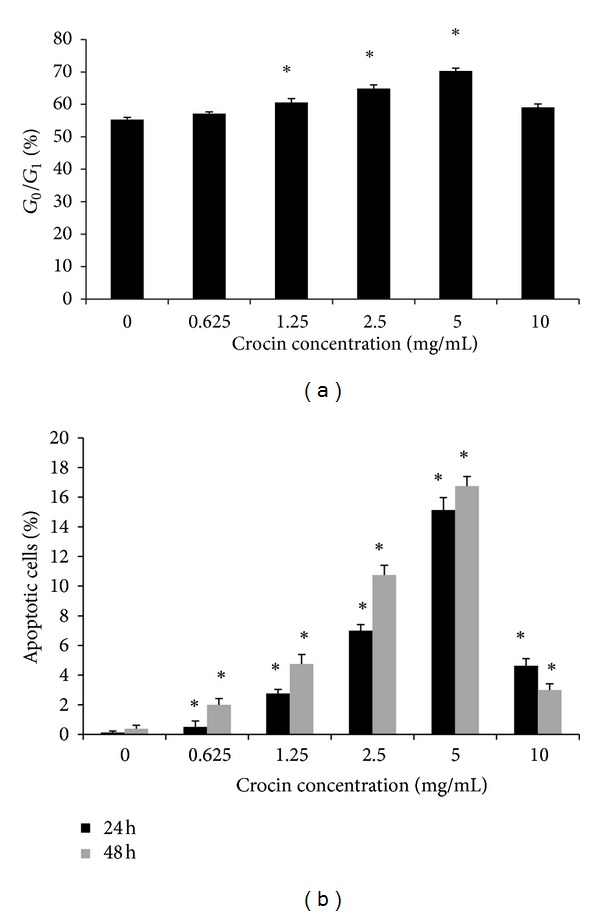
Crocin induces apoptosis and cell cycle arrest of HL-60 cells. (a) HL-60 cells were treated by crocin at the indicated concentration for 48 h, and the ratio of cells at G0/G1 was calculated based on flow cytometry. (b) HL-60 cells were treated by crocin at the indicated concentration for 24 or 48 h, and the percentages of apoptotic cells were calculated based on AO/EB staining. *P < 0.05 versus control.

To determine whether crocin inhibits the proliferation of HL-60 cells through the regulation of cell cycle progression and apoptosis, first we performed flow cytometry using PI staining. We observed a significant increase of G0/G1 cells from 55.33% in control group to 70.27% in the crocin-treated group (5.0 mg/mL). However, at 10 mg/mL, crocin could not further increase the cell ratio in G0/G1 phase (Figure 2(a)). These results suggest that crocin was capable of inducing cell cycle arrest at G0/G1. 

 AO/EB staining showed that uniformly green live cells with normal morphology were seen in the control HL-60 cells, whereas green early apoptotic cells with nuclear margination and chromatin condensation occurred in HL-60 cells treated by 0.625–2.5 mg/mL crocin, and orange later apoptotic cells with fragmented chromatin and apoptotic bodies were seen in HL-60 cells treated by 5 mg/mL. The percentage of apoptotic cell significantly increased gradually with crocin concentration increased from 0.625 to 5 mg/mL, compared with the control group, and the effects were time dependent (Figure 2(b)). However, at the concentration of 10 mg/mL, crocin induced cell necrosis rather than apoptosis. These results suggest that crocin could induce HL-60 cell apoptosis.

### 3.3. Antitumor Efficacy of Crocin In Vivo

**Figure 3:**
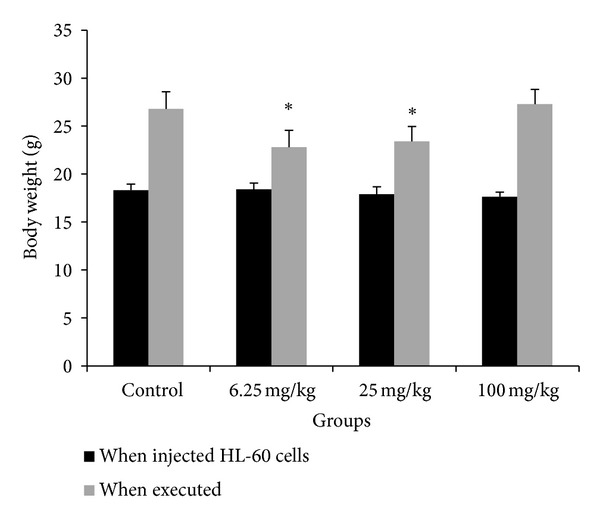
The body weight of mice that received HL-60 xenografts and crocin treatment. The body weight of mice was monitored daily throughout the experiment. Left panel: the body weight of mice at the beginning of receiving xenografts. Right panel: the body weight of mice after 28 days of crocin treatment. *P < 0.01 versus control.

After being injected HL-60 cells, spontaneous activity and food intake of all mice decreased. At the time of receiving HL-60 cells, there was no significant difference in the body weight between the four groups. As the tumors grew, all the mice's weight increased (Figure 3). There was no treatment-related death of mice.

 The tumor formation rate of the control and experiment groups (6.25, 25, 100 mg/kg crocin) was 100%, 50%, 75%, and 75%, respectively. There was no significant difference in the tumor formation rate among the four groups. The tumor formation time of the control and the experiment groups was 11.50 ± 1.60, 20.00 ± 1.15, 14.30 ± 1.86, and 10.50 ± 1.64 d, respectively. The tumor formation time of the experiment group (6.25 mg/kg) was obviously longer than the other three groups, and the tumor formation time of the experiment group (25 mg/kg) was longer than the control and experiment groups (100 mg/kg). These results suggest that crocin at the dose of 6.25 and 25 mg/kg could slow the formation of HL-60 cell xenograft in nude mice.

**Figure 4:**
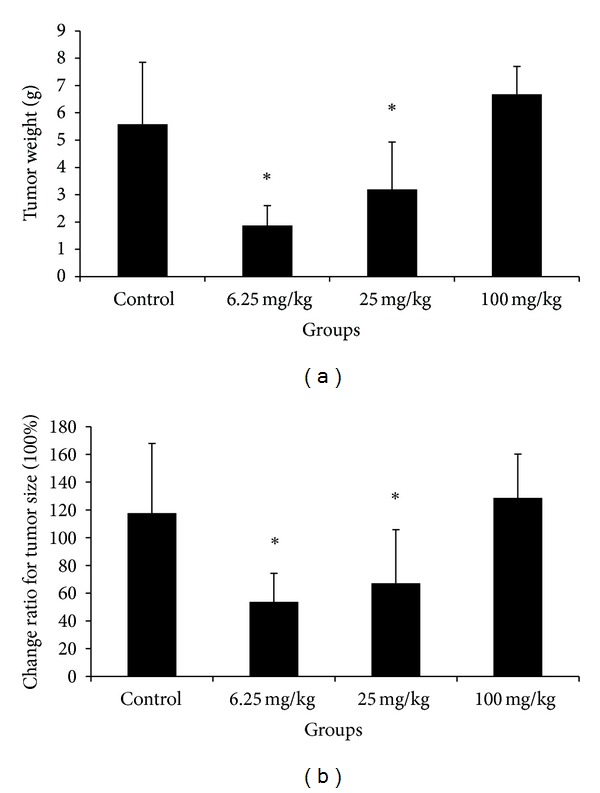
The tumor weight and size in mice that received HL-60 xenografts and crocin treatment. After 28 days of treatment, the mice were sacrificed, and the xenografts were excised. (a) Tumor weight in different treatment groups. (b) The change ratio of tumor size in different treatment groups. *P < 0.01 versus control.

 At the end of the study, the xenografts were excised from each sacrificed mouse, and tumor weight and volume were calculated. Tumor weight and the change ratio of tumor size in mice treated by crocin at the doses of 6.25 and 25 mg/kg were both significantly inhibited compared with the control group (Figures 4(a) and 4(b)). These results suggest that crocin could inhibit the growth of HL-60 cell xenograft in nude mice. 

**Figure 5:**
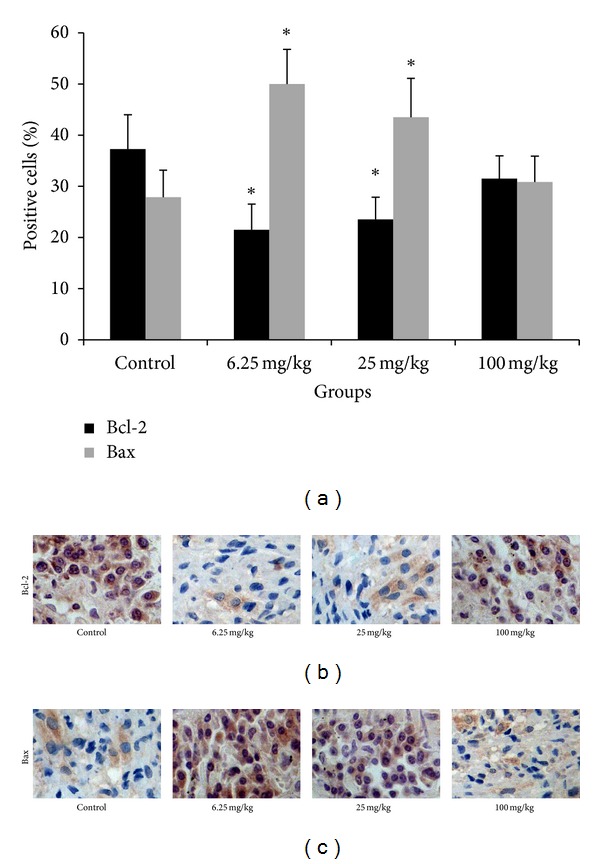
Immunohistochemical staining of Bcl-2 and Bax expression in HL-60 xenografts. (a) The percentages of cells stained positively for Bcl-2 and Bax in different groups. (b) Immunohistochemical staining of Bcl-2 in HL-60 xenografts from different groups. (c) Immunohistochemical staining of Bax in HL-60 xenografts from different groups. Magnification: 400x.

 To investigate whether the regression of tumor growth by crocin is due to the induction of apoptosis in vivo, we performed immunohistochemistry analysis of Bcl-2 and Bax expression in xenograft. The number of Bcl-2 positive cells was decreased in tumors from mice treated by 6.25 or 25 mg/kg crocin, compared to those from controls. In contrast, the number of Bax positive cells was increased in tumors from mice treated by 6.25 or 25 mg/kg crocin, compared to those from controls (Figure 5). 

**Figure 6:**
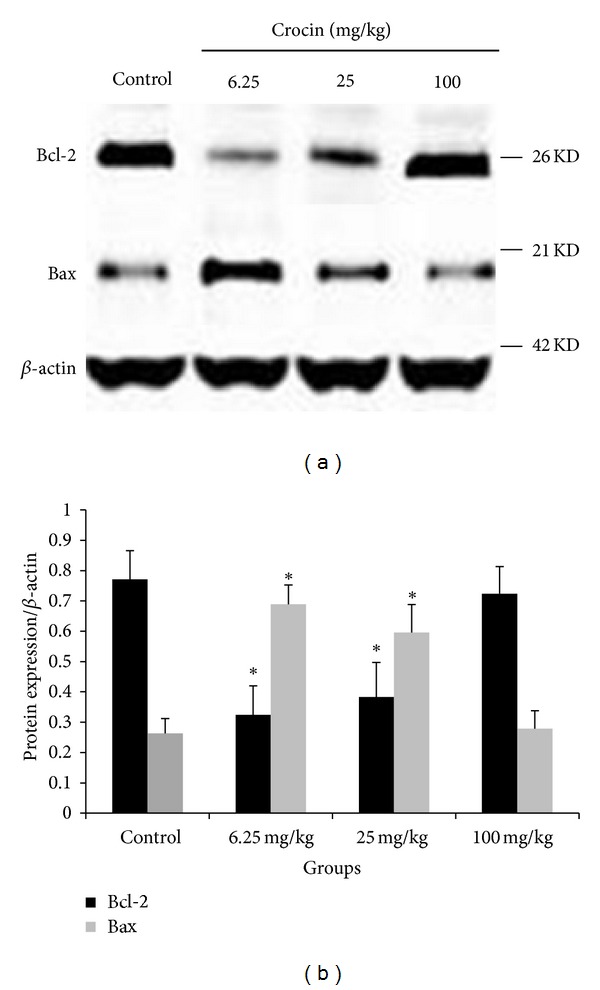
Crocin regulates the expression levels of Bcl-2 and Bax in HL-60 xenografts. The mice were treated with crocin (0, 6.25, 25, or 100 mg/kg, qd), and xenografts were collected for western blot analysis. Left panel: representative blots. Right panel: quantization of relative Bcl-2 and Bax protein levels in different groups. *β*-actin was used as loading control. *P < 0.05 versus control.

 We also performed western blot analysis of Bcl-2 and Bax expression in xenografts. The results showed that the protein level of Bcl-2 was reduced in tumors derived from mice treated with 6.25 or 25 mg/kg crocin, compared to those from control. In contrast, the protein level of Bax was increased in tumors derived from mice treated with 6.25 or 25 mg/kg crocin, compared to those from control (Figure 6). Taken together, these data indicate that crocin could reduce Bcl-2 expression and increase Bax expression, leading to increased apoptosis in HL-60 cell xenograft. 

## 4. Discussion

In the present study, we showed that crocin, a main compound derived from Crocus sativus extract, could inhibit the proliferation and induce the apoptosis of HL-60 cells both in vitro and in vivo. These data provide strong evidence that crocin has the potential for the treatment of leukemia.

 Anti-tumor drugs are known to regulate cell cycle progression, inhibit cell growth and proliferation, and induce apoptosis in tumor cells [8]. Crocin could induce the significant alteration of gene expression profile of T24 cell, and its anti-tumor effects have been proposed to be medicated at least in part by regulating the cell cycle progression [5]. Another study reported that crocin could induce apoptosis and G1-phase cell cycle arrest of human pancreatic cancer cell line [4]. In this study we showed that within the range of 0.625–5 mg/mL, crocin induced the apoptosis of HL-60 cells in a dose-dependent manner. However, higher dose of crocin at 10 mg/mL induced cell necrosis, suggesting the toxic effect of crocin at high dose. Similarly, we found that crocin at the dose of 0.625–5 mg/mL could induce G0/G1 phase arrest of HL-60 cells in a dose dependent manner. Collectively, these data suggest that crocin inhibits HL-60 cell proliferation by inducing G0/G1 arrest and apoptosis of HL-60 cells.

 To confirm our in vitro results, we employed nude mice xenograft model to evaluate the in vivo anti-tumor effects of crocin. Our results showed that crocin at the dose of 6.25, 25 mg/kg had strong inhibitory effect on HL-60 cell growth in nude mice, while the high dose (100 mg/kg) had no significant inhibitory effect, perhaps due to the toxic effects.

 There was no accidental death throughout the course of the animal experiment, indicating the safety of crocin. It was demonstrated that orally administered crocin was not absorbed in plasma either after a single dose or repeated doses, and crocin was excreted largely through the intestinal tract following oral administration [9]. Another study reported that crocin was not detected in blood plasma following oral administration [10]. In the present study, oral administration was not adopted, and we observed obvious anti-tumor effects of crocin after daily intraperitoneal injection in nude mice, indicating that crocin could be absorbed following intraperitoneal injection. 

 Medicinal herbs have been shown to exert anti-tumor effects by the induction of apoptosis in cancer cells including leukemia cells [11–13]. Bcl-2 and Bax are important antiapoptotic and proapoptotic molecules, respectively. Crocin suppressed TNF-*α* induced apoptosis of PC12 cells by modulating mRNA expression of Bcl-2 family proteins [14]. In this study, immunohistochemical and western blot analysis indicated that crocin at dose of 6.25 or 25 mg/kg could increase Bax expression while decreasing Bcl-2 expression. These results suggest that crocin inhibits tumor growth by modulating the expression of apoptosis-related molecules. However, further investigation is necessary to elucidate the molecular mechanism by which crocin regulates the expression of Bcl-2 and Bax.

## 5. Conclusions

In summary, both in vitro and in vivo studies demonstrate that crocin inhibits the proliferation and tumorigenicity of HL-60 cells, which may be mediated by the induction of apoptosis and cell cycle arrest and the regulation of Bcl-2 and Bax expression. These findings suggest that crocin has the potential to be developed as a new drug with high efficacy and low toxicity for the treatment of leukemia.
